# Electrocardiographic abnormalities in patients with heart failure

**Published:** 2008-02

**Authors:** Kamilu M Karaye, Mahmoud U Sani

**Affiliations:** Department of Medicine, Bayero University, Kano, Nigeria; Department of Medicine, Bayero University, Kano, Nigeria

## Abstract

**Background:**

The morbidity and mortality from heart failure (HF) differ between patients with reduced (< 50%) and with preserved (≥ 50%) left ventricular ejection fraction (LV EF) on account of many factors, including abnormalities detected in the electrocardiogram (ECG). The aim of this study was to determine and compare the ECG abnormalities between HF patients with reduced and with preserved LV EF.

**Methods:**

The study was cross-sectional in design and carried out in Aminu Kano teaching hospital and Murtala Mohammed specialist hospital, Kano, Nigeria, from April 2005 to June 2006. We studied the resting electrocardiograms of all HF patients aged 15 years and older who were referred to the two centres for echocardiography.

**Results:**

A total of 113 patients were studied and 98.2% of them had abnormal ECGs. Forty-two patients (37.2%) had preserved LV EF while the remaining 71 (62.8%) had reduced LV EF. Left ventricular hypertrophy (LV H) was the commonest ECG abnormality, found among 55 patients (77.5%) with reduced LV EF, and 21 patients (50%) with preserved LV EF (*p* = 0.0026). The commonest arrhythmia was atrial fibrillation, found among 10 patients (14.1%) with reduced LV EF and eight patients (19.1%) with preserved LV EF (*p* = 0.486). Prolonged corrected QT interval was found among 30 (71.4%) and 56 patients (78.9%) with preserved and reduced LV EF, respectively (*p* = 0.370).

**Conclusion:**

Most of the patients with heart failure studied in Kano, Nigeria had abnormal electrocardiograms, and the most common abnormality was LV H.

## Summary

The electrocardiogram (ECG) at rest is a non-invasive investigation that is recommended in the initial evaluation of patients with heart failure (HF).^1^ This is because the ECG is crucial in the detection of many abnormalities that may either cause or worsen HF.^1^

The syndrome of HF may present with reduced and/or preserved left ventricular ejection fraction (LVEF). Over the past few years, there has been a growing appreciation that a large number of patients with HF (20−60%) have ‘preserved LVEF’.^1^ Differences have been identified in the demography, morbidity and mortality of patients presenting with reduced or preserved LVEF.^1^ These differences are due to many factors, including those found on the ECG.

To the best of our knowledge, there is a paucity of data on ECG abnormalities in heart failure patients in many developing countries, including Nigeria, where this study was carried out. The aim of the study was therefore to determine and compare the ECG abnormalities among HF patients presenting with preserved (≥ 50%) and those with reduced (< 50%) LVEF in Kano, Nigeria.

## Patients and methods

The study was cross-sectional in design, conducted at the echocardiography centres of Aminu Kano teaching hospital (AKTH) and Murtala Mohammed specialist hospital (MMSH), both in Kano, Nigeria. These centres serve the populace of Kano and neighbouring states.

Before the commencement of this study, official approval for conducting the study was sought from the ethics committees of both AKTH and MMSH. The study conformed to the principles outlined in the Declaration of Helsinki on the ethical principles for medical research involving human subjects.^2^ All patients aged 15 years and older referred for echocardiography and who had HF gave their informed consent to participate in the study and were recruited consecutively. None of these patients was on treatment with anti-arrhythmic drugs. Data were collated over 15 months, from April 2005 to June 2006.

At the time of booking for echocardiography at the study centres, patients were routinely encouraged to bring their recent 12-lead resting ECG and chest radiograph on their appointment days. This was to assist the echocardiographer/cardiologist in making better-informed comments on the echocardiographic findings. This background provided the opportunity for us to recruit patients into the study, but we only used ECGs recorded after the diagnosis of HF was made.

About 95% of the ECGs were recorded by a trained clinical assistant using a Bionet Cardiocare EKG-2000 machine at AKTH, and the remaining 5% were recorded in other hospitals in Kano but were of good quality. All ECGs were recorded at the standard calibration of 25 mm/s.

To confirm the diagnosis of HF, a brief history was taken and a physical examination carried out. The definition of HF from the European Society of Cardiology was adopted.^3^ The ECGs at rest for all the recruited patients were studied and interpreted by the authors in the standard fashion.^4^ The Estes criteria^5^ and Morris index^6^ were adopted for the definitions of left ventricular hypertrophy (LVH) (point score ≥ 5) and left atrial enlargement (LAE), respectively. Correction of QT interval (QTc) was done using the Bazette formula.^7^ Standard definitions of other variables were adopted.^8^

All the echocardiograms were performed at each centre by the authors on a Toshiba diagnostic ultrasound machine (model SSA 325A), using a 3.75-MHz sector transducer. The procedure was performed and measurements taken according to the recommendations of the American Society of Echocardiography.^9^

Rheumatic mitral stenosis (MS) was defined by the presence of the following echocardiographic features: thickened (sometimes calcified) mitral leaflets and subvalvular apparatus, decreased E−F slope, ‘hockey-stick’ appearance of the anterior mitral leaflet in diastole, immobility of the posterior mitral leaflet, narrowed ‘fish-mouth’ orifice in the short-axis view measurable with planimetry, and increased LA size. Rheumatic mitral regurgitation (MR) was defined on the echocardiogram by the presence of thickened and retracted leaflets and subvalvular apparatus with poor co-aptation of the leaflets in systole, which could be worsened by the dilatation of the mitral annulus.^10^ Other rheumatic valvular lesions were defined according to standard criteria.^10^

Data were analysed using SPSS software version 10.0. The Chi-squared or Fisher’s exact tests were used to test for significance of observed associations between categorical variables, and the student’s *t*-test was used to compare means. A *p*-value of < 0.05 was considered significant.

## Results

A total of 113 patients were studied, out of which 42 (37.2%) were males and 71 (62.8%) females, giving a male:female ratio of 1:1.69. The mean age of all patients was 42.82 ± 18.31 years, with a range of 15−90 years.

A total of 42 patients (37.2%) had LVEF ≥ 50%, whereas 71 (62.8%) had LVEF < 50%. The mean LVEF was 64.57 ± 10.57% and 34.06 ± 8.05% for patients with preserved and reduced LVEF, respectively (*p* < 0.001). Females outnumbered males in both groups (64.3% in the preserved LVEF and 62.0% in the reduced LVEF groups) (*p* = 0.806). The mean age of patients with preserved LVEF (39.29 ± 18.86 years) was lower than that of subjects with reduced LVEF (44.92 ± 17.78 years), but the difference was not statistically significant (*p* = 0.115).

Various ECG abnormalities were found in 111 patients (98.2%), whereas ECGs in the remaining two patients (1.8%) were normal (at rest). The majority of patients (65.5%) had at least three ECG abnormalities, while 27.4% had two abnormalities and 5.3% had only one.

The corrected QT interval (QTc) was prolonged (defined as > 440 ms in males and > 460 ms in females)^8^ in 30 patients (71.4%) with preserved LVEF and in 56 patients (78.9%) with reduced LVEF (*p* = 0.370). Mean QTc in patients with LVEF ≥ 50% was 466.73 ± 41.68 ms, and 468.52 ± 34.16 ms in those with LVEF < 50% (*p* = 0.893). The other ECG abnormalities in the two groups of patients are presented and compared in Table 1.

**Table 1.** Electrocardiographic Abnormalities In Patients With Preserved And Reduced Left Ventricular Ejection Fraction

*Abnormalities*	*LVEF ≥ 50% n = 42 (%)*	*LVEF < 50% n = 71 (%)*	*Total n = 113 (%)*	p*-value*
LVH	21 (50.0)	55 (77.5)	76 (67.3)	0.0026*
LAE	13 (31.0)	45 (63.4)	58 (51.3)	0.001*
Sinus tachycardia	16 (38.1)	26 (36.6)	42 (37.2)	0.875
LAD	3 (7.1)	16 (22.5)	19 (16.8)	0.035*
AF	8 (19.1)	10 (14.1)	18 (15.9)	0.486
PVC	2 (4.8)	7 (9.9)	9 (8.0)	0.279
RAD	6 (14.3)	2 (2.8)	8 (7.1)	0.030*
ST-T wave abnormality	3 (7.1)	5 (7.0)	8 (7.1)	0.629
Complete LBBB	_	6 (8.5)	6 (5.3)	
Complete RBBB	5 (11.9)	1 (1.4)	6 (5.3)	0.026*
Minor IVCD	2 (4.8)	4 (5.6)	6 (5.3)	0.604
Low voltage complexes	3 (7.1)	2 (2.8)	5 (4.4)	0.266
BAE	3 (7.1)	1 (1.4)	4 (3.5)	0.144
AMI	1 (2.4)	2 (2.8)	3 (2.7)	0.690
Others	8 (19.1)	10 (14.1)	18 (15.9)	0.486

LVEF, left ventricular ejection fraction; *n*, number of patients; LVH, left ventricular hypertrophy; LAE, left atrial enlargement; LAD, left axis deviation; AF, atrial fibrillation; RAD, right axis deviation; LBBB and RBBB; left and right bundle branch block; IVCD, intra-ventricular conduction defect; BAE, bilateral atrial enlargement; AMI, acute myocardial infarction.

In addition, the following isolated abnormalities were identified among patients with reduced LVEF: bifascicular block, incomplete left bundle branch block (LBBB), ventricular and atrial bigeminy, couplets, indeterminate axis, atrial flutter, junctional rhythm and atrial tachycardia. Patients with preserved LVEF also had the following isolated abnormalities: right atrial enlargement (RAE), incomplete right bundle branch block (RBBB), atrial ectopics, couplets, indeterminate axis, atrial flutter and sinus bradycardia.

The aetiologies of HF in patients with preserved and with reduced LVEF are presented in Table 2. The aetiologies were similar in both groups, except for dilated cardiomyopathy (DCM), which was found exclusively in patients with reduced LVEF, and effusive pericarditis found only in patients with preserved LVEF. The predominant rheumatic valvular disease among all patients was mixed mitral valve (MV) disease found among 15 patients (13.3%) (10 of them had reduced LVEF, the remaining five patients had preserved LVEF), followed by rheumatic MR found among six patients (5.3%) (five of them had preserved LVEF). Pure rheumatic MS was found in only one patient who had reduced LVEF. Patients with rheumatic heart disease (RHD) had the largest mean LA dimension (53.81 ± 11.86 mm).

**Table 2.** Aetiology Of Heart Failure In Patients With Preserved And Reduced Ejection Fraction

*Diagnosis*	*LVEF ≥ 50% n = 42 (%)*	*LVEF < 50% n = 71 (%)*	*Total n = 113 (%)*	p*-value*
HHD	17 (40.5)	32 (45.1)	49 (43.4)	0.634
RHD	10 (23.8)	12 (16.9)	22 (31.0)	0.370
DCM	_	17 (23.9)	17 (15.0)	
PPHD	2 (4.8)	7 (9.9)	9 (8.0)	0.279
IHD	1 (2.6)	3 (4.2)	4 (3.5)	0.524
Effusive pericarditis	4 (9.5)	_	4 (3.5)	
Other	8 (19.1)	_	8 (7.1)	
Total	42 (37.2)	71 (62.8)	113 (100)	

LVEF, left ventricular ejection fraction; *n*, number of patients; HHD, hypertensive heart disease; RHD, rheumatic valvular heart disease; DCM, dilated cardiomyopathy; PPHD, peripartal heart disease; IHD, ischaemic heart disease.

The following diseases were also rare causes of HF among the patients with preserved LVEF: cor pulmonale, acute myocarditis, hypertrophic cardiomyopathy, alcoholic cardiomyopathy, thyrotoxicosis and tetralogy of Fallot.

## Discussion

ECG abnormalities were found in almost all the patients with HF (98.2%), and the majority (65.5%) of the patients had at least three such abnormalities. This finding is consistent with earlier reports from both within and outside Nigeria,^11^,^12^ and further affirms that a normal ECG is rare in heart failure patients.

The commonest ECG abnormality found in all patients, as well as in those with reduced or preserved LVEF was LVH. The next most frequent abnormality was LAE in patients with reduced LVEF, and sinus tachycardia in those with preserved LVEF. In comparison, the frequency of LVH and LAE were significantly higher in patients with reduced LVEF (*p* = 0.0026 and 0.001 respectively). Similarly, Opadijo and Omotosho^11^ reported that LVH was the commonest ECG abnormality, found in 68% of patients with HF and reduced LVEF. In contrast however, left axis deviation (LAD) was the next most common abnormality, whereas only two patients had LAE. In both studies, HHD was the most common aetiology of HF.

The reasons behind the disparities in the results might be related to differences in the criteria used for defining ECG abnormalities. In a study comparing ECG abnormalities between HF patients with preserved LVEF and those in whom it was reduced, Thomas *et al.*^13^ also reported that LVH, LAE and sinus tachycardia were more common in patients with reduced LVEF (*p* = 0.002, 0.001 and 0.004, respectively). Hypertension was similarly common among the studied patients, affecting 78 and 74% of patients with preserved and reduced LVEF, respectively.

From the foregoing, it appears that the high prevalence of hypertension in the discussed studies had dictated the main findings, with a preponderance of LVH. Hypertensive heart disease predisposes to the development of left ventricular hypertrophy, cardiac arrhythmia, heart failure, myocardial ischaemia, left atrial abnormalities and functional valvular regurgitation.^14^ The prognostic significance of LVH among hypertensive patients is well established. It is often considered the ‘haemoglobin A_1c_ of BP’, since it is an objective measure of both the severity and the duration of elevations of BP. Progressive LVH may lead to decreased LV compliance, decreased coronary reserve, ventricular ectopy and impaired systolic function.^14^

Other than HHD, the present study has revealed that RHD, mainly in the form of rheumatic mixed MV disease and MR, played an important role in the aetiology of LAE. Left atrial dilatation is a well-recognised complication of rheumatic mitral valve diseases.^10^

Atrial fibriallation (AF) was found to be the most common arrhythmia (affecting 16% of all patients; 19.1% of preserved LVEF group and 14.1% of reduced LVEF group; *p* = 0.486). Thomas *et al.*^13^ reported similar findings in HF patients with preserved LVEF (19%) and reduced LVEF (10%) (*p* = 0.09).

Opadijo and Omotosho^11^ also found AF in 7.3% of HF patients with reduced LVEF and a mean age of 57.3 years. In contrast, Owan *et al.*^15^ found AF in 28.5% of HF patients with normal LVEF and 41.3% of those with reduced LVEF (*p* < 0.001), while Bhatia *et al.*^16^ reported a prevalence of 23.6 and 31.8% (*p* < 0.001), respectively. The mean age of patients in the latter two studies was above 70 years. The three studies^13^,^15^,^16^ were all carried out in the United States and Canada. However, 75% of the patients studied by Thomas *et al.*^13^ were African-Americans and only 10% were Caucasians; the mean age of all the patients was 56.5 years.

In general, AF is more widespread in whites than blacks and in the elderly than in the young.^17^ Moreover, CAD was the commonest cause of HF in the studies by Owan *et al.*^15^ and Bhatia *et al.*,^16^ whereas HHD was the most frequent cause in our study and those by Thomas *et al.*^13^ and Opadijo and Omotosho.^11^

LAD was found in 16.9% of all patients, and was significantly more common in patients with reduced LVEF (*p* = 0.035). This is in contrast to the result obtained by Opadijo and Omotosho,^11^ who found LAD among 48.3% of all patients with reduced LVEF. Complete LBBB was found in 8.5% of patients with reduced LVEF and not in those with preserved LVEF. Complete LBBB was absent among the patients studied by Opadijo and Omotoso,^11^ but same group found it in 9% of hypertensive patients followed up over five years for cardiovascular morbidity and mortality.^18^ Left bundle branch block is an important finding in patients with heart failure, because of its association with worsening of HF symptoms and LV systolic function, as well as increased mortality.^19^

QTc was prolonged in 71.4 and 78.9% of HF patients with preserved and reduced LVEF respectively, but the difference was not statistically significant (*p* = 0.370). Vrtovec *et al.*^20^ also reported that 51% of patients with heart failure had prolonged QTc, while Boccalandro *et al.*^21^ reported that the mean QTc among HF patients was prolonged (mean of 447 ± 33 ms) and inversely related to the severity of HF. The proportion of HF patients with prolonged QTc in our study was as high, perhaps because the majority of them were females (62.8%) and most also had LVH (67.3%). The female gender^7^,^22^ and cardiac hypertrophy^23^ are factors among many others that independently prolong QTc.

Other ECG abnormalities were uncommon in heart failure patients, which was in agreement with an earlier report.^11^

An important limitation to our study was the lack of ambulatory ECG monitoring, which might have yielded more information than we have reported. Importantly, patients with a normal ECG at rest may develop abnormalities during physical activity. Unfortunately, this facility was not available in our study centres.

## Conclusion

We found that the ECG in HF patients was almost always abnormal, and most patients had at least three abnormalities. Left ventricular hypertrophy was the most common abnormality in all patients combined, as well as in the two patient groups. In addition, HF patients with LVEF < 50% had more ECG abnormalities.
